# Identification of promising dipeptidyl peptidase-4 and protein tyrosine phosphatase 1B inhibitors from selected terpenoids through molecular modeling

**DOI:** 10.1093/bioadv/vbae205

**Published:** 2024-12-19

**Authors:** Oludare M Ogunyemi, Gideon A Gyebi, Femi Olawale, Ibrahim M Ibrahim, Opeyemi Iwaloye, Modupe M Fabusiwa, Stephen Omowaye, Omotade I Oloyede, Charles O Olaiya

**Affiliations:** Structural and Computational Biology Group, Nutritional and Industrial Biochemistry Research Unit, Department of Biochemistry, College of Medicine, University of Ibadan, Ibadan 200005, Nigeria; Department of Biochemistry, Faculty of Science and Technology, Bingham University, New Karu, Nasarawa 961105, Nigeria; Department of Biochemistry, University of Kwazulu Natal, Durban 4041, South Africa; Department of Biophysics, Faculty of Sciences, Cairo University, Giza 12613, Egypt; Department of Biochemistry, Federal University of Technology, Akure 340110, Nigeria; Africa Centre of Excellence for Mycotoxin and Food Safety, Federal University of Technology Minna, Minna 920101, Nigeria; Department of Biosciences, Salem University, Lokoja, P.M.B. 1060, Nigeria; Department of Biochemistry, Ekiti State University, Ado-Ekiti, Ekiti State P.M.B. 5363, Nigeria; Structural and Computational Biology Group, Nutritional and Industrial Biochemistry Research Unit, Department of Biochemistry, College of Medicine, University of Ibadan, Ibadan 200005, Nigeria

## Abstract

**Motivation:**

Investigating novel drug–target interactions is crucial for expanding the chemical space of emerging therapeutic targets in human diseases. Herein, we explored the interactions of dipeptidyl peptidase-4 and protein tyrosine phosphatase 1B with selected terpenoids from African antidiabetic plants.

**Results:**

Using molecular docking, molecular dynamics simulations, molecular mechanics with generalized Born and surface area solvation-free energy, and density functional theory analyses, the study revealed dipeptidyl peptidase-4 as a promising target. Cucurbitacin B, 6-oxoisoiguesterin, and 20-epi-isoiguesterinol were identified as potential dipeptidyl peptidase-4 inhibitors with strong binding affinities. These triterpenoids interacted with key catalytic and hydrophobic pockets of dipeptidyl peptidase-4, demonstrating structural stability and flexibility under dynamic conditions, as indicated by dynamics simulation parameters. The free energy analysis further supported the binding affinities in dynamic environments. Quantum mechanical calculations revealed favorable highest occupied molecular orbital and lowest unoccupied molecular orbital energy profiles, indicating the suitability of the hits as proton donors and acceptors, which likely enhance their molecular interactions with the targets. Moreover, the terpenoids showed desirable drug-like properties, suggesting their potential as safe and effective dipeptidyl peptidase-4 inhibitors. These findings may pave the way for the development of novel antidiabetic agents and nutraceuticals based on these promising *in silico* hits.

**Availability and implementation:**

Not applicable.

## 1 Introduction

Diabetes mellitus (DM) ranks among the most challenging public health burdens in the world as it affects about 463 million (Kaur *et al.* 2021) and may increase to 552 million by the year 2030 and 700 million by 2045 as projected by the International Diabetes Federation (Whiting *et al.* 2011, Cho 2016, Cho *et al.* 2018, Kaur *et al.* 2021). It is generally characterized by hyperglycemia and impaired glucose homeostasis resulting from defects in insulin secretion, insulin action, or both (Reimann *et al.* 2009, Mourad *et al.* 2021). Type I diabetes mellitus (T1DM) is implicated in ∼10% of the diabetic population while Type II diabetes mellitus (T2DM) contributes ∼90% of diabetes-related cases (American Diabetes Association 2014). While T1DM is often managed by insulin treatments and lifestyle modifications (Piłaciński and Zozulińska-Ziółkiewicz 2014), an ideal treatment for T2DM is still elusive and many patients are still unable to meet their glycemic goals due to the intricate pathogenesis (Bhat *et al.* 2021, Rosenzweig and Sampson 2021). Therefore, the persistent unaddressed needs of most patients remain an important factor driving the quest for better prevention and treatment options for DM.

Drug targets of current relevance in diabetes have been exploited for the development of several promising therapeutic agents (Moller 2012, Kanwal *et al.* 2022). While many of the agents are promising oral agents for T2DM patients, they are not without various side effects. Examples include the starch blockers, insulin secretagogues, insulin mimickers, and insulin sensitizers (Kasole *et al.* 2019, Panigrahy *et al.* 2021, Kanwal *et al.* 2022, Ogunyemi *et al.* 2022). Acarbose, sulfonylureas, meglitinides, thiazolidinediones, biguanides aimed at blunting postprandial glucose rise, suppressing hepatic glucose output, stimulating insulin release, and increasing peripheral glucose utilization respectively are already approved for clinical use (He *et al.* 2015, Kaur *et al.* 2021), with reported adverse effects (Tahrani *et al.* 2011, Kaur *et al.* 2021). Beyond alpha-glucosidase, alpha-amylase, and other starch blockers widely exploited for antidiabetic drug development (Ogunyemi *et al.* 2020), the dipeptidyl peptidase-4 (DPP-4) enzyme is an emerging drug target and has been recommended in the treatment of diabetes as it acts in carbohydrate metabolism by delaying gastric emptying, increasing insulin secretion, and reducing glucagon secretion (Nauck 2016, Kanwal *et al.* 2022). The DPP-4 inhibitors stimulate the breakdown of endogenous incretin hormones and promote pancreatic glucose-dependent insulin secretion. The enzyme is a serine peptidase which acts by rapidly degrading the incretin hormones, such as glucagon-like peptide 1 (GLP-1) that play an important role in blood glucose regulation, causing short life span of the enzyme. Thus, a therapeutic strategy that involves inhibiting DPP-4 would help to maintain the endogenous level of GLP-1 and increased β cell mass; and thereby enhance glucose-dependent insulin secretion, slow down gastric emptying, and reduce postprandial glucagon and food intake (Koliaki and Doupis 2011, Salvo *et al.* 2016, Paternoster and Falasca 2018). Metformin is the first-choice medication which is most frequently recommended for treating T2D. This drug acts by improving glucose metabolism through both AMP-activated kinase activation and enhanced GLP-1 release (Glossmann and Lutz 2019). Thus, it improves insulin sensitivity of relevant tissues (Rena *et al.* 2017, Biondani and Peyron 2018). However, metformin like other antidiabetic drugs is often accompanied by moderate to severe side effects (McGovern *et al.* 2018). Another emerging target for enhancing insulin sensitivity in various cells is the protein tyrosine phosphatase 1B (PTP1B). By improving the sensitivity of the insulin receptor, PTP1B inhibitors have the potential to cure insulin resistance-associated ailments (McGovern *et al.* 2018, Teimouri *et al.* 2022). The PTP1B acts by negatively regulating insulin receptor signaling pathway in cells (Verma *et al.* 2017). Agents that inhibit this enzyme can activate phosphorylation of several insulin receptor kinase substrates which in turn stimulate the phospho-inositide-3-kinase as well as subsequent protein kinase B expression. This further promotes translocation of glucose transporter into the cell membrane from intracellular vesicles; and thereby promote uptake of glucose molecules by the cells for energy generation. Due to the major roles played by insulin disturbance in diabetes, insulin signaling is highly recommended for targeting diabetes and explored for developing new antidiabetic agents (Rosenzweig and Sampson 2021).

African flora possesses a very rich biodiversity of herbs, spices, and medicinal which is widely exploited in traditional, alternative, and complementary medicine for treating several diseases including DM (Kasole *et al.* 2019, Mohammed and Tajuddeen 2022). Most traditional healers in Africa who detain the ancestral heritage of these medicinal plants are illiterate with their ethnopharmacological knowledge transmitted verbally from generation to generation which is at risk of disappearing. Thus, the World Health Organization (WHO) has recommended scientific studies to document the folk knowledge and validate the acclaimed therapeutic potential of these plants from the perspective of developing improved medications (Panigrahy *et al.* 2021). In this direction, the literature is replete with scientific reports on the antidiabetic activity of African herbs, spices, and medicinal as previously revealed by excellent review articles (Mohammed *et al.* 2014, 2017, Mohammed and Tajuddeen 2022). Most of these reported studies mainly focused on plant extracts and/or fractions, with only few having got scientific validation. Several ethnopharmacologically important antidiabetic herbs in Africa have shown potent activity against DPP-4 (Mohammed and Tajuddeen 2022). The extracts of *Antidesma madagascariense* Lam, whose leaf and stem bark decoctions are used in folk medicine in Madagascar for the management of DM, showed inhibitory activity against DPP-4 enzyme with an IC_50_ value of 79.2 mg/ml (Mahomoodally *et al.* 2015, Beidokhti *et al.* 2018). This inhibitory potential indicates the ability of the extract to improve insulin sensitivity. In the same vein, several bioactive constituents of African indigenous herbs have been screened through several *in vitro* and *in vivo* studies for activity against PTP1B. Ethyl acetate fraction and isolated pimarane diterpenoids from *Icacina oliviformis*, a popular food spice native to the regions of West and Central Africa, have been reported to inhibit PTP1B (Catarino *et al.* 2016, Zhou *et al.* 2020, Mohammed and Tajuddeen 2022). Mohammed and Tajuddeen (2022) revealed that the widely reported African antidiabetic plants contain several classes of compounds which include terpenoids, alkaloids, flavonoids, and carotenoids. Owing to their wealth of chemical structures, such antidiabetic plants are considered as an inventory of bioactive compounds which might be useful as a basis for drug discovery and development in combating diabetes.

Terpenoid structures, which belong to an important class of natural products offer exciting possibilities for the development of potent nutraceuticals and drug candidates as they are widely reported for antidiabetic activities (Silva *et al.* 2016, Panigrahy *et al.* 2021). For example, compounds like oleanolic acid and ursolic acid, found in various food herbs and medicinal plants including holy basil and rosemary, have shown potential in improving glucose metabolism and insulin sensitivity in animal studies (Silva *et al.* 2016). Several drug-like compounds derived from such terpenoid structures are already under various stages of preclinical and clinical evaluation geared toward developing antidiabetic agents (Panigrahy *et al.* 2021, Rosenzweig and Sampson 2021). The antidiabetic potential of plant-derived terpenoids could be attributed to varying mechanisms of action which include: increasing insulin sensitivity (Chang *et al.* 2015, Panigrahy *et al.* 2021), inhibiting pancreatic amylase and glucosidase enzymes (Majouli *et al.* 2016, Panigrahy *et al.* 2020), reducing oxidative stress (Gong *et al.* 2012), and inhibiting the development of diabetic complications (Ma *et al.* 2019, Kumar *et al.* 2020). Several studies have revealed that terpene-rich bioactive extracts of several African medicinal plants have shown antidiabetic activity by targeting key proteins associated with type II diabetes *in vitro* and *in vivo* as reviewed by Panigrahy *et al.* (2021). Several terpene-rich plant extracts and terpene isolates have been widely reported to target DPP-4 (Purnomo *et al.* 2015), PTP1B (Liang *et al.* 2013, Wu *et al.* 2014), and other targets (Panigrahy *et al.* 2021) in diabetes *in vitro* and *in vivo*.

Molecular modeling tools are valuable for identifying novel drug–target interactions (DTIs), which characterize the binding of drug compounds to their specific targets. The binding affinity of drugs which is a measure of the strength of the DTI greatly influences the drug’s efficacy and therapeutic potential. Modeling methodologies including structure predictions, target-ligand docking, molecular mechanics (MM) and quantum mechanics computations, molecular dynamics (MD) simulation, and free energy calculations can provide an accurate and efficient theoretical description of a biomolecular system, providing useful insights into DTIs which are often difficult to reach or unreachable through experiments (Olawale *et al.* 2022a; Ogunyemi *et al.* 2023). Leveraging the sample space minimization and resource maximization capacity of such methodologies, the molecular targets of uninvestigated phytochemicals can be explored rapidly and more economically providing great insights into their biological activities. When such tools are integrated, the 3D visualization of the electronic and steric molecular properties of the interaction between bioactive compounds and drug targets could be computed in atomistic details enabling more targeted experimental and preclinical evaluations. Earlier studies have revealed the potential of *in silico* methods for predicting DPP-4 inhibitors (Kaur *et al.* 2018, Chalichem *et al.* 2021, Quek *et al.* 2021) and PTP1B inhibitors (Olawale *et al.* 2022b; Quy *et al.* 2022) from natural sources. As reviewed in this paper, African herbs with cornucopia of secondary metabolites have a long history of antidiabetic activity as indicated by their folkloric use and empirical evidence. However, the bioactive components that may account for the therapeutic role are largely unexplored. Screening the bioactive compounds against emerging drug targets helps to expand the chemical space coverage of the targets. Therefore, the study focused on exploring the interactions of the active sites of DPP-4 and PTP1B with 107 terpenoids from African antidiabetic plants using molecular docking, MD simulation, MM-GBSA, and density functional theory (DFT) calculations.

## 2 Methods

### 2.1 Preparation of protein structures

The 3D structure of human DPP-4 (PDB ID: 3G0B) which was co-crystallized with T22800 (alogliptin) and that of the human PTP1B (PDB ID: 2CM7), which was co-crystalized with isothiazolidinone, were retrieved from the Protein Data Bank (https://www.rcsb.org). From these protein structures, the co-crystalized compound and water molecules were deleted. Each hydrogen atom was integrated into protein structures using Autodock Vina 4.2 program.

### 2.2 Ligand preparation

An in-house library of 107 terpenoids previously reported from African medicinal plants was created after a thorough search of the literature. The structures of the terpenoids from the in-house library and the protein native ligands were obtained in the structural data format (SDF) from the PubChem database at www.pubchem.ncbi.nlm.nih.gov. Discovery studio was used to convert SDF structures of ligands and the standard drugs into PDB chemical format. The ChemDraw version 19 was used to prepare unavailable structures, which were then converted to mol2 chemical format and then to PDB chemical format. As previously demonstrated, the nonpolar hydrogen molecules were combined with the carbon atoms, and the polar Gasteiger-type hydrogen charges were assigned to the atoms in the chemical structures (Gyebi *et al.* 2021). Internal degrees of freedom and torsions were both set to 0. The structures were then transformed using AutoDock Tools into the dockable PDBQT format.

### 2.3 Molecular docking calculations

A docking algorithm based on the Larmackian Genetic Algorithm (GA) was used to assess the binding tendency of the selected terpenoids with the target proteins. The structures of the terpenoids were imported through OpenBabel (O’Boyle *et al.* 2011) into AutoDock Vina incorporated in PyRx 0.8 (Trott and Olson 2010). Energy minimization of the ligands was performed using universal force field with conjugate gradient descent as the optimization algorithm. The structures of the terpenoids were then docked with the binding pockets of both DPP-4 and PTP1B. The active site regions of these enzymes were defined and selected using grid boxes with parameters depicted in Table 1. While the exhaustiveness was set as 8, other GA parameters including population size, number of generations, crossover rate, mutation rate, elitism, local search, and local search iterations were set as default. After the scoring, the docked poses were retrieved and the ligand–protein interactions visualized using Discovery Studio Visualizer version 16.

**Table 1.** Grid box parameters for defining active site regions of DPP-4 and PTP1B.

Dimensions	DPP-4 (Å)	PTP1B (Å)
center_x	34.88	14.79
center_y	29.17	−3.40
center_z	16.00	1.69
Size x	21.92	16.50
Size y	17.77	19.72
Size z	22.83	27.64

### 2.4 Prime MM-GBSA postdocking calculations

Postdocking free energy simulation through MM with generalized Born and surface area solvation (MM-GBSA) was applied to the top ligand–protein complexes in order to validate the docking score. To achieve this, the MM-GBSA panel in Maestro was employed to compute the binding affinity of the top terpenoids with the target enzymes as demonstrated in a previous study (Olawale *et al.* 2023). The MM-GBSA helped to estimate the difference in free binding energy between the terpene structures and the enzymes in both the nonbound state and complexed form after the process of energy minimization. The OPLS3forcefield parameters were used for the MM-GBSA, while VSGB was employed to model the continuum solvent. The computations were done with all other options set as default. The binding free energy computations were undertaken based on the following equations:


(1) $$\Delta G_{\mathrm{bind}}=\Delta E+\Delta G_{\mathrm{solv}}+\Delta G_{SA}$$

(2) $$\Delta E=E_{\mathrm{complex}}-E_{\mathrm{Enzyme}}-E_{\mathrm{Ligand}}$$

(3) $$\Delta G_{\mathrm{solv}}=\Delta G_{\mathrm{solv}\;(\mathrm{complex})}-\Delta G_{\mathrm{solv}\;\left(\mathrm{Enzyme}\right)}-\Delta G_{\mathrm{solv}\;(\mathrm{Ligand})}$$

(4) $$\Delta G_{SA}=\Delta G_{SA\;(\mathrm{complex})}-\Delta G_{SA\;\left(\mathrm{Enzyme}\right)}-\Delta G_{SA\;(\mathrm{Ligand})}$$

where

E is the minimized energies for molecular systems.

$\Delta G_{SA}\;$ represents the nonpolar contribution to the energy of solvation resulting from the surface area, while $G_{SA}$ is the surface energy of the systems.

### 2.5 MD simulation

Initially, the human DPP-4 (PDBID: 3G0B) protein was subjected to a 100-ns full atomistic MD simulation in order to generate an ensemble of the protein for performing the ensemble docking analysis. Then, the apo enzyme, and the DPP-4–terpene complexes obtained from docking calculations were subjected to MD simulation. These were performed using GROMACS 2019.2 and GROMOS96 43a1 selected as forcefield on the WebGRO (Oostenbrink *et al.* 2004, Abraham *et al.* 2015). The preliminary topology files of the terpene structures were prepared using PRODRG webserver (http://davapc1.bioch.dundee.ac.uk/cgi-bin/prodrg) (Schüttelkopf and van Aalten 2004). The apo DPP-4 and the DPP-4–terpene complexes were solvated within a cubic box of the transferable intermolecular potential using a four-point water model. The periodic boundary conditions were applied and a physiological condition of 0.154 M concentration set by neutralized NaCl ions. The minimization of the apo DPP-4 system and the complex systems was carried out in 10 000 steps employing the steepest descent algorithm and applying the NVT ensemble for 0.3 ns. This was followed by 0.3 ns of equilibration under NPT condition. The system temperature was set up and maintained at 310 K with the velocity rescale. The system pressure was also set and maintained at 1 atm employing the Parrinello-Rahmanbarostat. The integrator used for the computation was the Leap-frog integrator with a time step of 2 fs. During the 100-ns full atomistic MD simulation production run, 0.1-ns snapshot was saved with a total of 1000 frame for each biomolecular system. Various thermodynamic parameters viz: RMSD, root mean square fluctuation (RMSF), surface accessible surface area (SASA), radius of gyration (RoG), and hydrogen bond number were computed from the trajectory files obtained from the dynamic simulation runs using VMD TK console scripts (Humphrey *et al.* 1996).

### 2.6 Clustering analysis

The trajectory files obtained from the initial MD simulation of the DPP-4 protein was clustered to generate the ensemble of the protein which were used for the ensemble docking simulation. Also, the trajectory files obtained from the simulation runs of DPP-4–terpene complex systems were clustered. The clustering analysis was performed employing the elbow method using TTClust V 4.9.0. Following cluster generation, a representative structure of each cluster of the complexes was chosen for interaction analysis with the aid of the Protein–Ligand Interaction profiler (PLIP) (Salentin *et al.* 2015).

### 2.7 Binding free energy calculation using MM-GBSA

The Gmx_MMPBSA algorithm was used to calculate the binding free energy for each complex using Molecular Mechanics Generalized Born Surface Area. The salt concentration and the solvation method were 0.154 and 5, respectively. The internal and external dielectric constants were set to 1.0 and 78.5, respectively, with other options as default. The decomposition of free energy was calculated to find which amino acids within 10 Å contribute most to the binding (Miller *et al.* 2012, Valdés-Tresanco *et al.* 2021). The MM-GBSA approach utilized is depicted in Equation (5):


(5) ΔG = <Gcomplex—Greceptor—Gligand>

The “< >” indicates the means of the free energies of complex, protein, and terpene structures over the frames used for the calculations. Several frames (200) were used to calculate the free energy. Various energy terms were computed using Equations (6) to (10) as follows:


(6) ΔGbind=ΔH—TΔS 

(7) ΔH=ΔEgas+ΔEsol

(8) ΔEgas=ΔEele+ΔEvdW

(9) ΔEsolv=EGB+ESA

(10) ESA=γ.SASA

where:

Δ*H* represents the enthalpy which is computed from gas-phase energy and solvation-free energy (Esol). The TΔS term represents the entropy contributions to the total binding affinity which was not included because of the aim of comparing the relative binding free energies. Egas comprised the electrostatic and van der Waals energy terms; Eele, EvdW, respectively. Esol was computed from the polar solvation energy (EGB) and nonpolar solvation energy (ESA) which was estimated from the solvent-accessible surface area (Xue *et al.* 2018, Tuccinardi 2021).

### 2.8 Density functional theory

On the basis of the electron density associated with the ligands, DFT asserts that ground state energy and other molecular attributes are determined only by this density. As a basis set for a DFT calculation using the B3LYP functional and 6-31G** as a single-point energy calculation, the Jaguar panel of Maestro was employed in this work. A variety of molecular reactivity indicators, including electrophilicity, hardness, softness, electron affinity, ionization potential, and molecular electrostatic potential, were assessed. The following mathematical equations were used to perform the computations as discussed previously (Elekofehinti and Iwaloye 2021).


(11) Electron affinity EA≈-LUMO

(12) $$\mathrm{Ionization potential}\;\left(\mathrm{IP}\right)\approx -\left(\text{HOMO}\right)$$

(13) $$\mathrm{Hardness}\;\left(\eta \right)\approx \left(\frac{\mathrm{IP}-\mathrm{EA}}{2}\right)$$

(14) $$\mathrm{Softness}\;\left(\sigma \right)\approx \left(\frac{1}{2n}\right)$$

(15) $$\text{Ell}\mathrm{ectrogativity}\;\left(\varkappa \right)\approx \left(\frac{\mathrm{IP}+\mathrm{EA}}{2}\right)$$

(16) $$\text{Chemical}\;\text{potential}\;\left(µ\right)=-\left(I+A\right)/2$$

(17) $$\text{Electrophilicity}\;\left(\omega \right)={µ}^{2}/2\eta$$

### 2.9 Physicochemical and admetSAR analysis

The hit triterpene structures were passed through predictive physicochemical analysis. The drug-like properties were obtained from the SwissADME web server platform (http://www.swissadme.ch/index.php) (Daina *et al.* 2017). The ADMETox prediction was performed on the Protox-II webserver (https://tox-new.charite.de/protox_II/index.php)(Banerjee *et al.* 2018). To perform these analyses, the SDF file format as well as the canonical SMILES of the of the selected terpenoids was retrieved from the PubChem Database and imported into the various webservers for druglikeness and ADMET computations using default parameters.

## 3 Results

### 3.1 Binding affinity and interactions of terpenoids with DPP-4 enzyme

The initial screening of 107 terpenoid structures against DPP-4 enzyme based on the Larmackian Genetic Algorithm docking protocol revealed 37 compound structures with docking scores (−7.7 to −10.0 Kcal/mol) lower or comparable to that of the co-crystalized alogliptin (−7.7 Kcal/mol) (Table S1). Ranking based on the docking scores and screening for favorable interactions with the target enzyme revealed the top seven compounds (Table 2), most of which belong to the triterpenoids subclass of terpenoids.

**Table 2.** Docking scores of terpenoids against DPP-4.

S/N	Compounds	Class	Dockingscore (Kcal/mol)	PostDocking MM-GBSA (Kcal/mol)
S1	Alogliptin		−7.7	−37.02
T1	Cucurbitacin B	Tetracyclic triterpenoids	−9.9	−47.80
T2	20-*Epi*-isoiguesterinol	Bisnortriterpenoids	−9.9	−29.78
T3	Isoiguesterin	Bisnortriterpenoids	−9.7	−28.75
T4	6-Oxoisoiguesterin	Bisnortriterpenoids	−9.3	−32.90
T6	Isoiguesterinol	Bisnortriterpenoids	−9.0	−29.36
T7	1-Deacetylkhivorin	Limonoids	−8.9	−19.72
T8	7-Deacetylkhivorin	Limonoids	−8.8	−25.71

**Figure 1.**
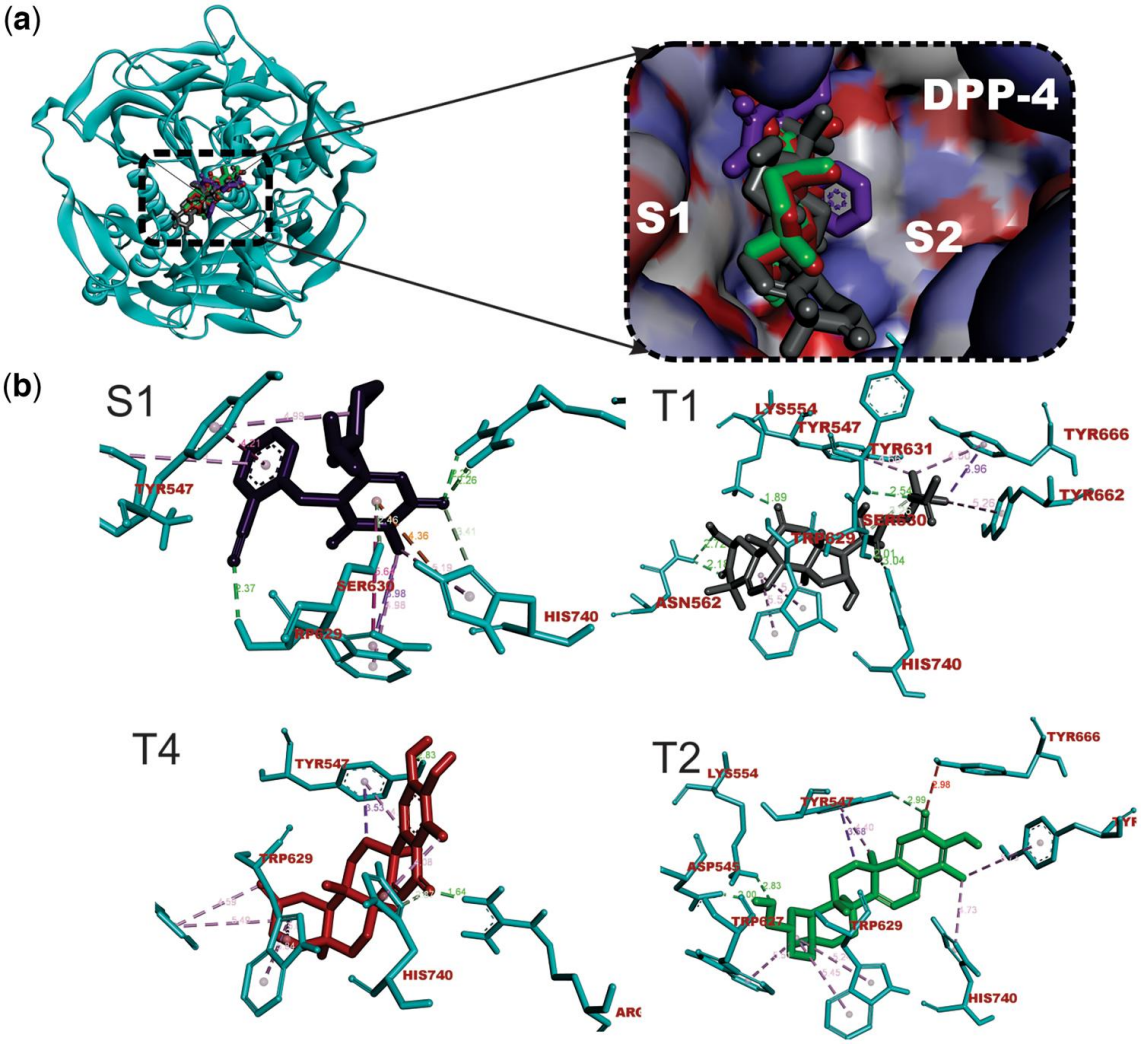
(A) Cartoon and surface views of the DPP-4-ligand interactions (B) The 3D representation of the interactions of hit triterpenoids (T1—Cucurbitacin B, T2—20-Epi-isoiguesterinol, T4—6-oxoisoiguesterin) and alogliptin (S1) with amino acid residues in the active site of DPP-4. Ligand structures are depicted in stick representations. Three-letter codes were used to represent amino acid residues.

The topmost seven terpene-DPP-4 complexes were subjected to postdocking MM-GBSA computations. While MM-GBSA (ΔGbind) of the reference alogliptin was −37.02Kcal/mol, the MM-GBSA (ΔGbind) of the top-docked terpene structure ranged from −47.80 kcal/mol (Cucurbitacin B) to −19.72 kcal/mol (1-Deacetylkhivorin) as shown in Table 1. It is worthy of note that Cucurbitacin B with the lowest binding energy with DPP-4 also exhibited the lowest MM-GBSA free binding energy to the enzyme. While van der Waals, coulombic, and nonpolar solvation energies made the most notable free energy contributions to Cucurbitacin B binding to DPP-4, van der Waals and nonpolar solvation made important contributions to binding of several other terpenoids (Table S2). The terpene structures demonstrated high levels of binding affinity, suggesting exceptional potential for sustained interactions with the DPP-4 enzyme. Interaction analysis of the selected terpenoids with the binding pocket of DPP-4 revealed that cucurbitacin B, 6-Oxoisoiguesterin, and 20-Epi-isoiguesterinol had binding poses with DPP-4 similar to that of the co-crystalized alogliptin. These compounds also featured strong and favorable interactions with the critical amino acid residues in the active site of the enzyme in a similar manner to the native compound as shown in Table 3. Table 3 shows that the hit triterpenoids and alogliptin had strong interactions with various residues involved in the catalytic triad, oxyanion cavity, hydrophobic S1 pocket, and charged S2 pocket in the binding pocket of DPP-4. The 3D representations of the interactions are depicted in Fig. 1.

**Table 3.** Amino acid interactions of DPP-4 with top-docked terpenoids.

Compounds	Hydrogen bonds (Bond length Å)		Hydrophobic interaction		Other interactions	
	No	Residues	No	Residues	No	Residues
Alogliptin	5	ARG125(2.22; 2.26) TRP629(2.37) **HIS740(3.41) SER630(2.46)**	7	TRP629(3.99; 5.64; 4.98) TYR547(4.21; 4.99) **HIS740(5.19)** LYS554	1	**HIS740**
T1	7	LYS554 ASN562 **SER630** TYR631 **HIS740**	6	TYR666 TYR547 TRP629TYR662	0	None
T4	3	ARG125 TYR547 **HIS740**	7	TYR547 TRP627 TRP629 **HIS740**	0	None
T2	3	TYR547 LYS544 ASP545	7	TYR547 TRP627 TRP629TYR662 **HIS740**	0	None

NB: The catalytic residues are presented in bold font.

### 3.2 Binding affinity and interactions of terpenoids with PTP1B enzyme

**Figure 2.**
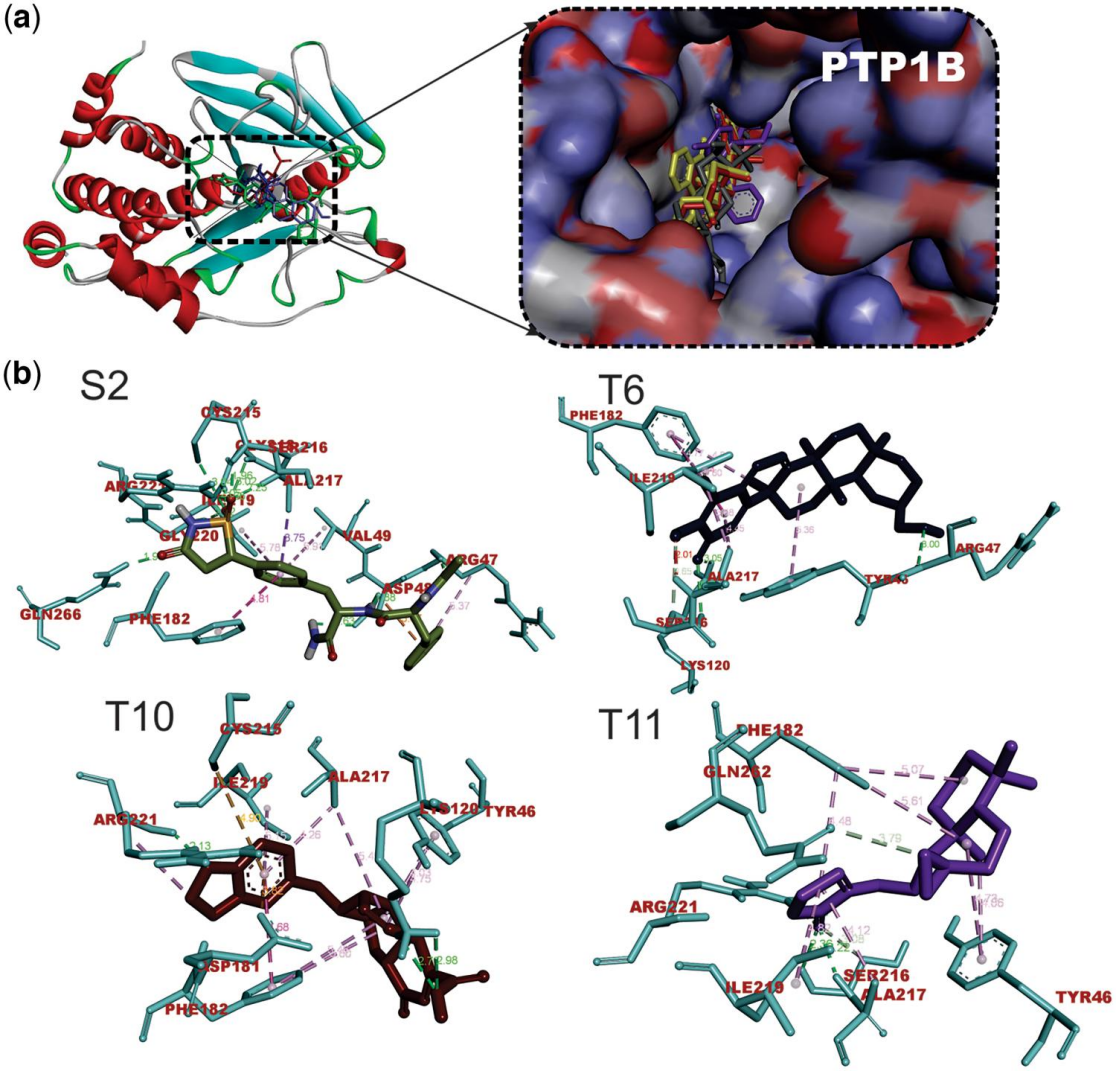
(A) Cartoon and surface views of PTP1B-ligand interactions. (B) The 3D representation of the interactions of hit triterpenoids and isothiazolidinone with critical residues in the binding pocket of PTP1B. The terpenoids are isoiguesterinol (T6), Cryptobeilic acid C(T10), Galanolactone (T11), and isothiazolidinone (S2). Ligand structures are depicted in stick representations. Three-letter code was used to represent amino acid residues.

The top 37 compounds obtained from docking interactions with DPP-4 were further screened against PTP1B using molecular docking, MM-GBSA postdocking analysis, and molecular interactions. Although all the docked compounds had docking scores and MM-GBSA binding energy higher than those of the reference isothiazolidinone, the topmost compounds had favorable interactions with the binding pocket of the protein. The molecular docking scores and prime MM-GBSA scores of the top terpenoids with PTP1B are shown in Table 4. Favorable interactions were also observed upon visualization of the top terpene–PTP1B complexes as shown in Table 5 and Fig. 2.

**Table 4.** Molecular docking and prime MM-GBSA binding energy of top terpenoids with PTP1B.

S/N	Compounds	Class	Dockingscore (Kcal/mol)	Prime MM-GBSA (Kcal/mol)
S2	Isothiazolidinone		9.8	−85.23
T9	Tsangibeilin B	Beilshmiedic acid derivatives	−8.4	−42.32
T10	Cryptobeilic acid C	Beilshmiedic acid derivatives	−8.1	−44.09
T3	Isoiguesterin	Bisnorterpenoids	−7.5	−42.07
T4	6-Oxoisoiguesterin	Bisnorterpenoids	−7.4	−41.11
T6	Isoiguesterinol	Bisnorterpenoids	−7.0	−45.82
T11	Galanolactone	Clerodane and labdane diterpenoids	−6.5	−43.56
T2	20-*Epi*-isoiguesterinol	Bisnorterpenoids	−6.2	−29.06

**Table 5.** Amino acid interactions of PTP1B with top hit terpenoids.

Compounds	Hydrogen bonds (Bond length Å)		Hydrophobic interaction (Bond length Å)		Other interactions (Bond length Å)	
	No	Residues	No	Residues	No	Residues
S2	11	ARG47(2.01) **CYS215(3.04) SER216(1.96)** ALA217(2.25) GLY218(3.02) ILE219(2.36) GLY220(2.02) **ARG221(2.05) GLN266(1.99) ASP48(1.88; 2.63)**	3	ALA217(3.75) PHE182(4.81) ARG47(5.37)	1	**ASP48(3.87)**
T6	4	ARG47 LYS120 SER216	7	PHE182(3) ALA217 ILE219 TYR46	0	None
T10	4	LYS120(2.76; 2.98) **ARG221(2.13; 2.06)**	8	PHE182(4.68; 5.45) ALA217(5.49; 4.26) **ARG221(3.74)** TYR46(4.75; 4.03) ILE219(5.15)	2	ASP181 (3.82) **CYS215(4.90)**
T11	5	**SER216(2.36; 3.08)** ALA217(2.22) **ARG221(2.28) GLN262(3.79)**	6	ALA217(4.12) ILE219(4.82) TYR46(4.73; 4.06) PHE182 (5.07; 4.48)	0	None

NB: The residues that define the catalytic triad are presented in bold font.

### 3.3 Docking analysis of DPP-4 conformers against top compounds

**Figure 3.**
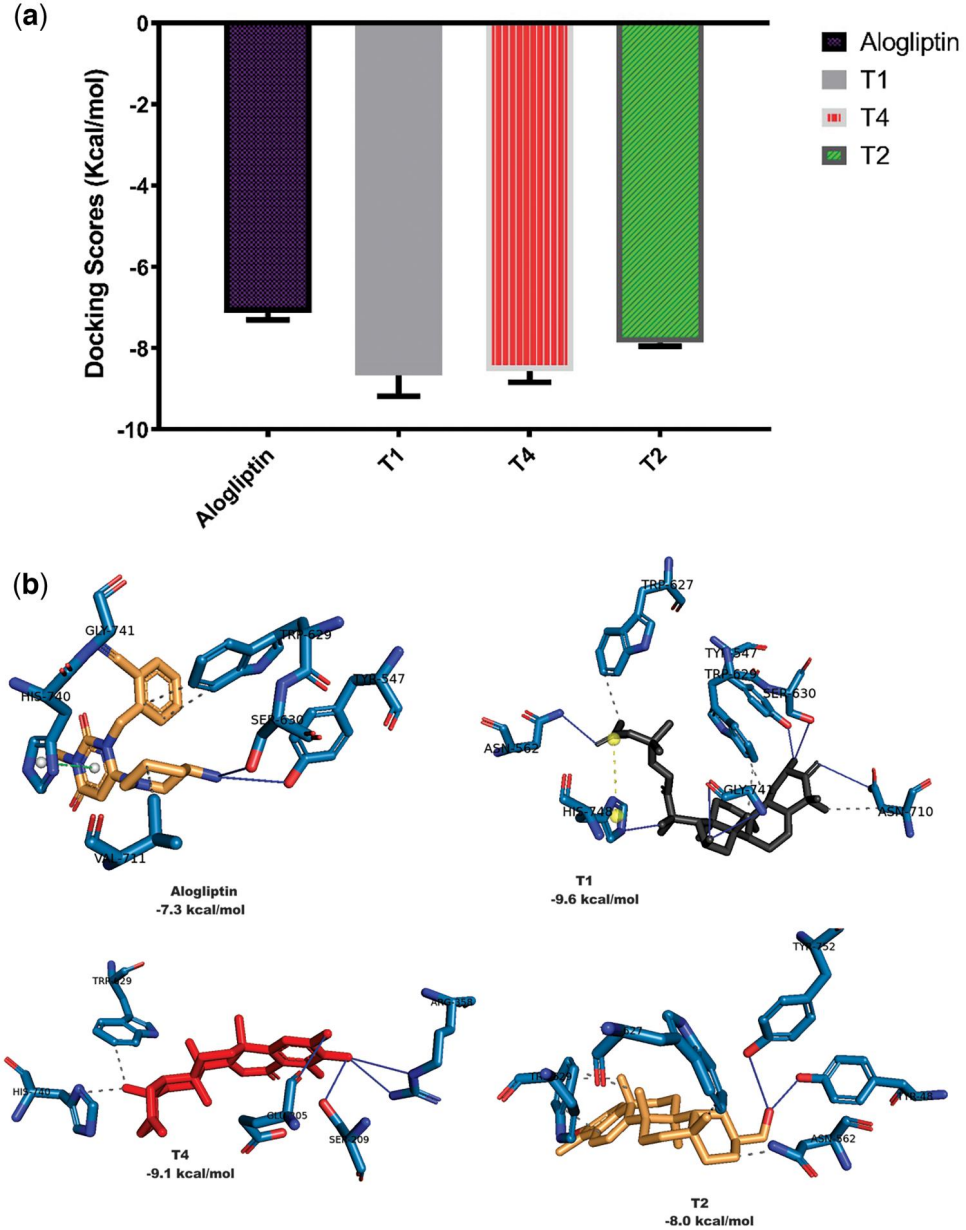
(A) Mean scores of docking of top triterpenoids (T1—Cucurbitacin B, T2—20-Epi-isoiguesterinol, T4—6-Oxoisoiguesterin) and alogliptin with structure ensemble of DPP4 (*n* = 4, scores are presented as mean ± SEM). (B) Interactions of top terpenoids T1, T2, and T4 with a representative conformer of DPP4.

Based on the results from molecular docking and postdocking MM-GBSA which revealed DPP-4 as a more promising target, three different coordinates of DPP-4 resulting from clusterization of the MD simulation trajectories were docked against the selected top three terpenoids (T1, T4, T2) along with the reference compound (alogliptin) using AutoDock Vina software (Morris *et al.* 2009, Trott and Olson 2010). The selected terpene structures were docked following the docking protocol. The docking complexes were analyzed using PLIP web server and visualized using PyMOL 2.4 software (Salentin *et al.* 2015). The ensemble docking analysis revealed that the average docking scores of the hit triterpenoids were lower than that of the reference alogliptin as shown in Fig. 3A. The 2D structures of the top triterpenoids are shown in Fig. 3B.

### 3.4 MD of DPP-4–ligand complexes

**Figure 4.**
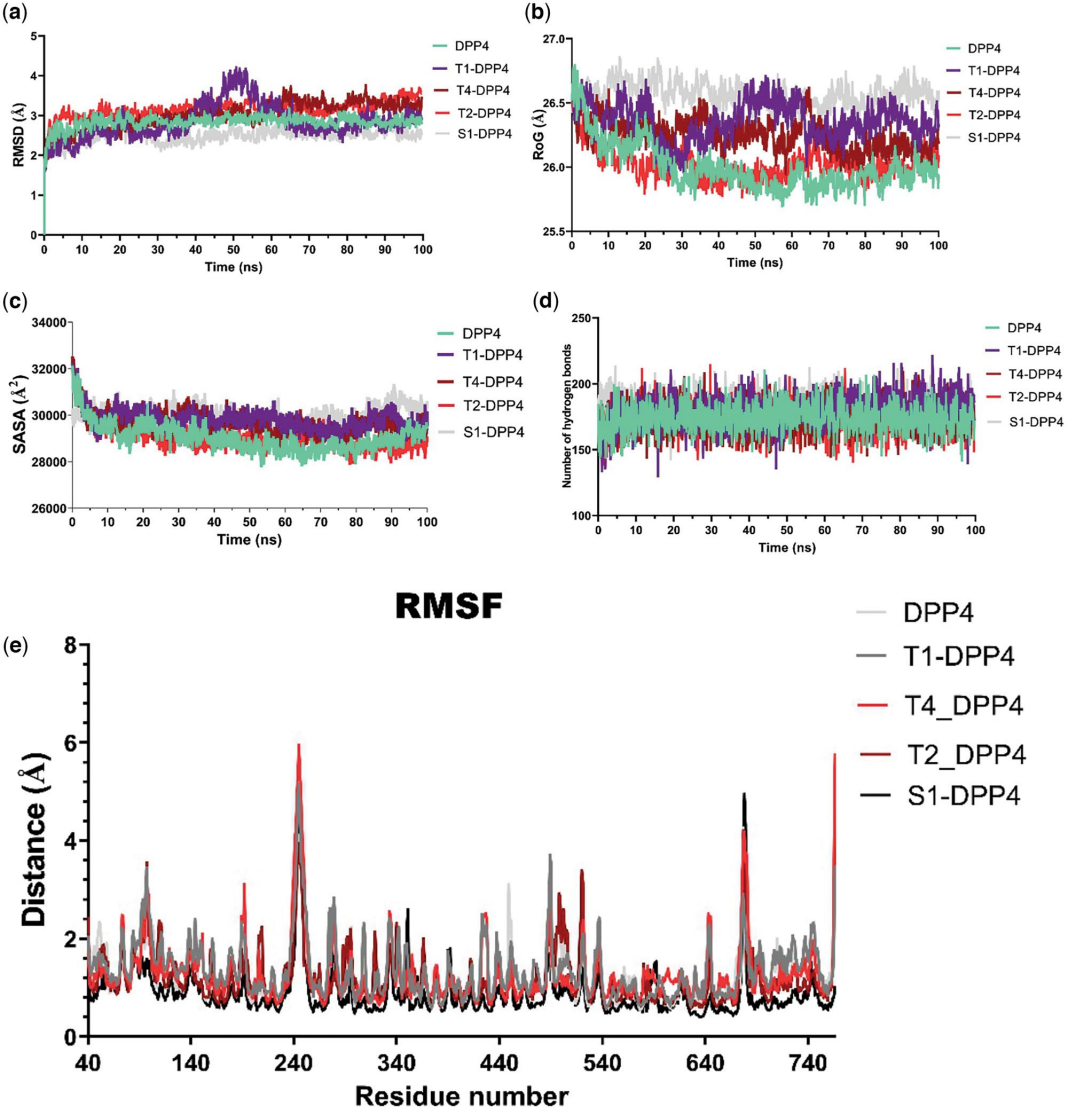
Molecular dynamics simulations analysis of DPP4 and its complexes with ligands. (A) Root mean square deviation (RMSD) of the backbone atoms over 100-ns simulation for DPP4 and its complexes with T1—cucurbitacin B, T2—20-Epi-isoiguesterinol, T4—6-Oxoisoiguesterin, and alogliptin (S1) ligands. (B) Radius of Gyration (RoG) representing the compactness of DPP4 and its ligand-bound complexes during the simulation. (C) Solvent accessible surface area (SASA) indicating the degree of protein surface exposure for DPP4 and the complexes. (D) Number of hydrogen bonds observed in DPP4 and its complexes throughout the simulation. (E) Root mean square fluctuation (RMSF) per residue illustrating the flexibility of DPP4 residues in the presence and absence of ligands. Each metric provides insights into the structural stability, compactness, surface exposure, intermolecular interactions, and dynamic behavior of DPP4 upon ligand binding.

The dynamics simulation was employed to model the interactions of the top-docking terpenoids with DPP-4 in a dynamic environment. The thermodynamic parameters that indicate the stability of the DPP-4 enzyme back-bone C-α atoms in complex with T1, T4, and T2 were calculated and plotted as shown in Fig. 4. The RMSD, a measure of the difference between two sets of coordinates, was computed and depicted in Fig. 4A.

The RMSD plot (Fig. 4A) revealed that the DPP-4 backbone, T1-DPP-4, T4-DPP-4, T2-DPP-4, and S1-DPP-4 molecular systems showed a slight rising RMSD trend in the initial 0.6, 0.8, 0.1, 0.6 and 1.1 ns and then stabilized at mean values of 2.84, 2.85, 3.02, 3.17 and 2.50 Å, respectively. These values indicated that the terpene–DPP-4 complexes reached a stable conformational trend over 100 ns of the simulation with few conformational transitions as the RMSD values were maintained within 2 Å. Except for the major fluctuations observed with the trajectory of DPP-4–cucurbitacin B complex, all the complexes exhibited relatively stable atomic trajectories displaying minor and consistent fluctuations. Figure 4B shows that the average RoG values for DPP-4 backbone, T1-DPP-4, T4-DPP-4, T2-DPP-4, and S1-DPP-4 biomolecular systems are 26.00, 26.36, 26.25, 26.02, and 26.57 Å, respectively. The results also revealed that the RoG values were maintained within 2 Å. The result shown in Fig. 4C revealed that the SASA values of DPP-4 backbone, T1-DPP-4, T4-DPP-4, T2-DPP-4, and S1-DPP-4 biomolecular systems are 29 052 Å^2^, 29 797 Å, 29 669 Å, 29 115 Å^2^, and 30 015 Å^2^, respectively. To provide more insight into the stability of the complexes, the number of H bonds found in the ligand–protein complexes was computed as depicted in Fig. 4D. The results revealed a steady trend for the five biomolecular systems which span around 173, 180, 174, 172, and 184 bonds for the DPP-4 backbone, T1-DPP-4, T4-DPP-4, T2-DPP-4, and S1-DPP-4 bio-molecular systems, respectively. The plot demonstrates large variations in the amino acid residues between 220 and 260 as well as those around 680, with the RMSF reaching notable peaks. Other residues, in addition to these also feature some notable peaks.

### 3.5 Free energy simulation of DPP-4–terpene complexes

To further validate the docking scores of the ligands to the protein, the trajectories obtained from the MD simulations of the terpene–DPP-4 complexes were subjected to free energy simulation using MM-GBSA. Table 6 shows a number of solvation and MM parameters and their energy contributions.

**Table 6.** The MM-GBSA calculations for DPP-4–triterpene complex MD trajectory.

Energy terms (Kcal/mol)	T1	T4	T2	S1
ΔVDWAALS	−34.67 ± 3.39	−22.20 ± 3.38	−30.73 ± 5.27	−22.63 ± 4.15
ΔEEL	−18.69 ± 7.22	−7.25 ± 12.28	125.46 ± 28.01	−304.75 ± 33.46
ΔEGB	46.29 ± 6.49	25.02 ± 10.43	−93.17 ± 30.7	312.75 ± 28.86
ΔESURF	−4.79 ± 0.43	−2.82 ± 0.65	−3.82 ± 0.66	−3.28 ± 0.53
ΔGGAS	−53.35 ± 7.49	−29.44 ± 13.02	94.73 ± 31.83	−327.39 ± 33.19
ΔGSOLV	41.5 ± 6.35	22.2 ± 10.09	−96.99 ± 30.24	309.48 ± 28.53
ΔTOTAL	−11.85 ± 3.34	−7.24 ± 4.62	−2.26 ± 3.64	−17.91 ± 6.42

**Figure 5.**
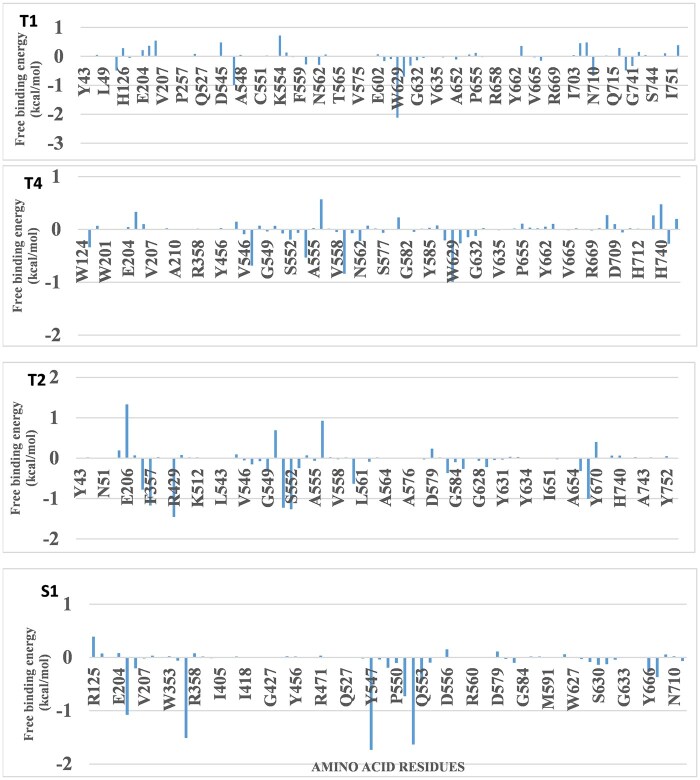
Free binding energy decomposition of key residues in DPP4 complexes. Energy decomposition analysis of binding interactions of DPP4 with T1—Cucurbitacin B, T2—20-Epi-isoiguesterinol, T4—6-Oxoisoiguesterin, and alogliptin (S1) ligands shows the per-residue free binding energy contributions (kcal/mol). T1 Complex highlights key residues contributing to the binding of ligand T1. T4 Complex displays the energetic contributions of residues in the binding of ligand T4. T2 Complex illustrates the binding energy contributions of residues interacting with ligand T2. S1 Complex shows the energetic contributions of residues involved in binding ligand S1. The analysis emphasizes the critical amino acid residues for ligand interaction and stability, revealing specific residues that favorably or unfavorably contribute to ligand binding affinity.

The results show a number of solvation and MM parameters and their energy contributions. Among the terpenoids, T1 (ΔGtotal = −11.85 Kcal/mol) exhibited the strongest binding affinity as indicated by the lowest free binding energy as compared to other terpenoids but with higher free binding energy than the reference agloliptin. The results also revealed that the main components of the free energy of the top complexes are van der Waals energy and electrostatic energy. Amino acids occurring around the binding pocket showed significant contributions to the overall binding affinity to the terpen–DPP-4 complexes as indicated by the MM-PBSA free energy decomposition analysis (Fig. 5).

### 3.6 Frontier molecular orbitals of top triterpenoids

To further investigate the behavior of the selected terpene structures as DPP-4 inhibitors, DFT computation was performed. Table 7 shows that the hit compounds had close band gaps which were quite low.

**Table 7.** Quantum chemical properties of DPP-4–ligand complexes.

Properties (eV)	S1	T1	T4	T2
HOMO	−0.20306	−0.24354	−0.24676	−0.24692
LUMO	0.06017	0.04816	0.05232	0.05377
Band gap	0.26323	0.2917	0.29908	0.30069
Electronegativity	0.071445	0.09769	0.09722	0.096575
Ionization potential	0.20306	0.24354	0.24676	0.24692
Electron affinity	−0.06017	−0.04816	−0.05232	−0.05377
Hardness	0.131615	0.14585	0.14954	0.150345
softness	3.798959	3.42818	3.343587	3.325684
Chemical potential	−0.07145	−0.09769	−0.09722	−0.09658
Electrophilicity	0.002552	0.004772	0.004726	0.004663

**Figure 6.**
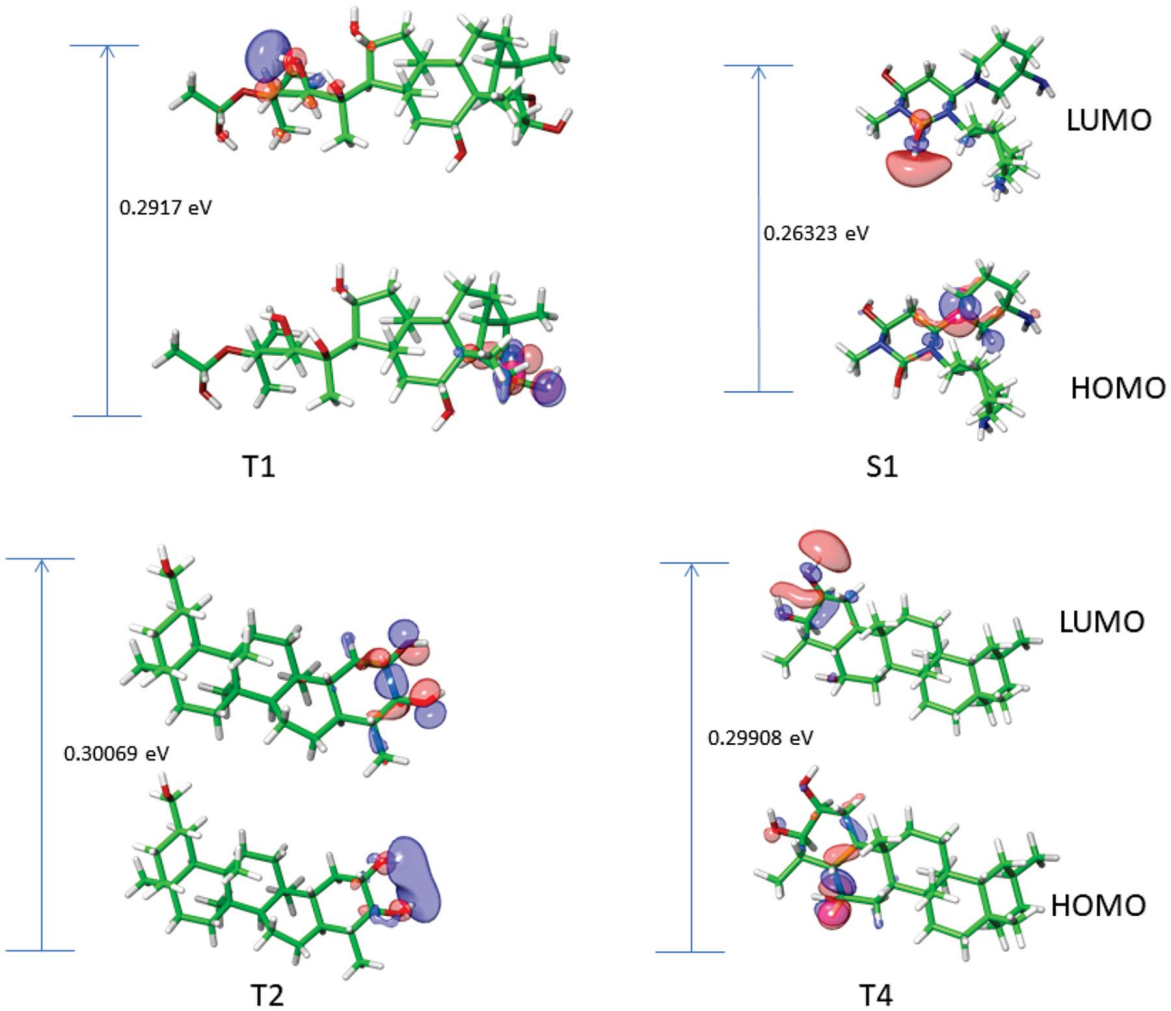
Molecular orbital images and calculated bandgap of top triterpene structures. The orbital stands for the likelihood of discovering an electron. The HOMO orbital appears to be centralized on the phenyl rings of the hit compounds while the LUMO orbital was predominantly on the side chains of the aromatic ring structures.

The HOMO and LUMO orbital of the hit compounds are given in Fig. 6. The regions of the compounds with red and blue color denote the positive and negative signal/phase of the molecular orbital, respectively.

### 3.7 Predicted physicochemical and ADMET properties of hit triterpenoids

The drug-likeness characteristics of the top triterpenoids were assessed to ascertain the suitability of the compounds as drug candidates. The drug-likeness characteristics of the compounds revealed desirable drug-like properties (Table 8). Using the Lipinski rule of five assessments, it was shown that all the hit compounds are in accordance to Lipinski rule.

**Table 8.** Physicochemical and ADMET characteristics of the hit triterpenoids.

Terpenoids	T1	T2	T4
MW	558.7	422.6	420.58
#Rotatable bonds	6	1	0
#H-bond acceptors	8	3	3
#H-bond donors	3	2	2
TPSA	138.2	57.53	57.53
ESOL Class	MS	MS	PS
Lipinski violations	1	1	1
Ghose violations	3	1	1
Veber violations	0	0	0
Egan violations	1	1	1
Muegge violations	1	1	1
Bioavailability Score	0.55	0.55	0.55
PAINS alerts	0	0	1
Brenk alerts	2	0	2
Synthetic Accessibility	6.79	6.29	5.21
GI absorption	Low	High	High
BBB permeant	No	No	No
Pgp substrate	Yes	No	No
CYP1A2 inhibitor	No	No	No
CYP2C19 inhibitor	No	No	No
CYP2C9 inhibitor	No	Yes	Yes
CYP2D6 inhibitor	No	No	No
CYP3A4 inhibitor	Yes	Yes	Yes
Bioavailability Score	0.55	0.55	0.55

S—Soluble; MS—Moderately Soluble; PS—Poorly Soluble.

## 4 Discussion

Investigating novel DTIs is crucial for expanding the chemical space of emerging therapeutic targets including DPP-4 and PTP1B in human diseases. The current study explored the interactions of active sites of DPP-4 and PTP1B with selected terpenoids derived from African antidiabetic plants using various molecular modeling techniques. The structure-based virtual screening of DPP-4 against 107 terpenoid structures through molecular docking helped to identify 37 top terpenoids with higher binding affinity tendency than the reference alogliptin. Postdocking MMGBSA analysis of the top terpenoids also revealed good binding affinity scores. It is worthy of note that Cucurbitacin B (T1) with the strongest binding affinity with DPP-4 as indicated by the lowest docking score also exhibited the lowest postdocking MM-GBSA free binding energy score to the enzyme. Molecular docking, MM-GBSA postdocking analysis, and molecular interactions also revealed the top terpenoids as potential inhibitors of PTP1B enzyme. Terpenoids, a class of compounds with compact molecular structure and less symmetry are known to possess ability to bind multifarious drug targets to exhibit therapeutic potential in human diseases (Panigrahy *et al.* 2021).

The interactions of the top terpenoids were analyzed alongside the reference drugs. The results showed that the interaction of the co-crystalized compound (alogliptin) with the binding site of DPP-4 involves several types of bonds including conventional hydrogen bonds, carbon–hydrogen bonds, Pi-cation electrostatic interactions, Pi-donor hydrogen bond, Pi-sigma hydrophobic interactions, Pi–Pi stacked hydrophobic interactions, and Pi-alkyl hydrophobic interactions. Key interactions of the co-crystalized compound involve carbon–hydrogen bond, Pi-cation electrostatic interactions, and Pi-alkyl hydrophobic interaction with HIS740 as well as Pi-donor hydrogen bond with SER630. The DPP-4 enzyme, which is catalytically active as dimers in both the soluble and cell-surface form, has 766 amino acids, containing a C-terminal hydrolase domain as well as the N-terminal propeller domain and a C-terminal hydrolase domain. In addition to the catalytic triad comprising Ser630, Asp708, and His740, DPP-4 contains Tyr47 and Ser631 associated with the oxyanion hole; Tyr631, Val656, Trp659, Tyr662, Tyr666, and Val711 functioning in the hydrophobic S1 pocket; and Arg125,

Glu205, Glu206, Phe357, Ser209, and Arg358 in the and a charged S2 pocket (Zhang *et al.* 2011, Antony *et al.* 2022). Interaction analysis of the selected terpenoid structures with the binding pocket of DPP-4 revealed that cucurbitacin B, 6-Oxoisoiguesterin (T4), and 20-Epi-isoiguesterinol (T2) had binding poses with DPP-4 similar to that of the co-crystalized alogliptin. Cucurbitacin B is a tetracyclic triterpene containing the cucurbitane nucleus skeleton, namely, 19-(10-9beta)-abeo-10alpha-lanost-5-ene with a several oxygen substitutions in different positions. It was observed that the oxygen substitutions in the structure played key roles in the interaction of the compound with the active site and hydrophobic S1 pocket in the binding site region of DPP-4. There was strong interaction with the catalytic triad residues as important carbonyl oxygen in the compound structure received a strong conventional hydrogen bond from the side chains of SER630 and another from HIS740 side chain. Also, the ether oxygen received a strong conventional hydrogen bond from the side chain of TYR631 in the hydro-phobic S1 pocket. In this pocket, the side chain of TYR666 conducted a Pi-sigma hydrophobic interaction with a methyl group and Pi-alky hydrophobic interaction with another methyl group in the compound. The side chain of TYR662 also conducted a Pi-alkyl hydrophobic interaction with a methyl group in the compound. This study suggests important roles of various van der Waals interactions, coulombic interactions, and nonpolar solvation energies in the binding interactions of Cucurbitacin B and the other hit compounds with DPP-4 enzyme. Coulombic interactions arise from the attraction or repulsion between charged particles. In the context of drug binding, these interactions can involve interactions between charged functional groups on the drug molecule and complementary charged residues in the binding site of the target protein. For example, positively charged drug molecules may form electrostatic interactions with negatively charged residues such as aspartate or glutamate, while negatively charged drug molecules may interact with positively charged residues such as lysine or arginine. Coulombic interactions contribute to the overall stability of the drug–receptor complex and can significantly influence binding affinity. The Van der Waals forces are attractive forces between atoms or molecules that arise from fluctuations in electron distribution. These interactions play a crucial role in drug binding as they contribute to the complementarity between the drug molecule and its binding site on the target protein. When a drug molecule fits into the binding site of its target receptor, van der Waals interactions help in maximizing the contact between the drug and the receptor by allowing close packing of atoms and stabilization of the complex. Optimizing van der Waals interactions is important for achieving high binding affinity and specificity. Nonpolar solvation energy refers to the energy associated with the solvation of nonpolar molecules or regions of molecules in a solvent. In drug binding, nonpolar solvation energy plays a role in the desolvation process, where water molecules surrounding the binding site need to be displaced to allow the drug molecule to bind. Hydrophobic interactions, driven by nonpolar solvation energy, are important for stabilizing the drug–receptor complex, especially in hydrophobic pockets of the binding site. Optimizing nonpolar solvation energy helps in enhancing the hydrophobic interactions between the drug and the receptor, thereby improving binding affinity. Cucurbitacin B, a flowering perennial plant that belongs to the family Cucurbitaceae, was derived from *Cogniauxia podolaena* (Banzouzi *et al.* 2008). It grows primarily in the wet tropical biome. The genus Cogniauxia is native to Angola, Cabinda, Cameroon, Central African Republic, Congo, Equatorial Guinea, Gabon, Guinea, Zaïre. Several other species in this genus are known to possess antidiabetic activity (Olarewaju *et al.* 2021).

Four bisnorterpenoids derived from *Ekebergia capensis* (Murata *et al.* 2008) viz: 20-Epi-isoiguesterinol, isoiguesterin, 6-oxoisoiguesterin, and isoiguesterinol were also observed to show stronger binding affinity than alogliptin as indicated by lower binding energies. The *Ekebergia capensis* is a flowering plant which belong the family Meliaceae is fondly called Cape ash and its range extends from the South African Eastern Cape to Sudan and Ethiopia. Its antidiabetic potential has been exploited in Eastern Cape KwaZulu-Natal, Limpopo, and Mpumalanga (Nyakudya and Tshabalala 2020). The herbal formulations comprising infusion prepared from the leaves of this plant and taken orally have shown antidiabetic activity streptozotocin-induced diabetic rats (Nyakudya and Tshabalala 2020). The 6-Oxoisoiguesterin (T4), one of the compounds from this plant interacted with the active site of DPP-4 through a carbon–hydrogen bond and Pi-alkyl hydrophobic interaction with HIS740. As shown in Table 2 and Fig. 1, 20-Epi-isoiguesterinol (T3), another bisnortriterpene, was observed to interact with the DPP-4 catalytic triad (HIS740) through a Pi-alkyl hydrophobic interaction. As extensively reviewed by Hamid *et al.* (2015), tetracyclic triterpenoids, a class of triterpenoids found in a variety of medicinal plants are notable for treating diabetes and its complications. In a previous study, botulin, a triterpene derived from samples of the root bark of Euclea undulate widely exploited traditionally to treat diabetes and other ailments in Southern Africa, has been reported to show a reduction in level of blood sugar as compared to reference drug glibenclamide (Hamid *et al.* 2015, Maroyi 2017). Our study provides insight into the molecular mechanism underpinning the activity of such drugs. Also, earlier studies on the structure–activity relationship of terpenoids have revealed the few roles of the molecular structure of terpenoids in manifesting their antidiabetic activity. For instance, Chen *et al.* (2019) revealed that sesquiterpenoids are highly effective as antidiabetic agents which may result from their structurally compact configuration, low ramification, and low symmetry. Another author revealed that the triterpenoids with hydroxyl and carboxyl functional groups exhibit high antidiabetic activity which is mediated through various mechanisms (Nazaruk and Borzym-Kluczyk 2015). In the review by Panigrahy *et al.* (2021), terpene structures containing the most hydrogen bonds and hydrophobic interactions exhibit enhanced antidiabetic activity. Other compounds with high affinity for DPP-4 are also reported in this study (Table 1). These include several acyclic triterpenoids derived from *Glossocalyx brevipes* (Siparunaceae) (Mbah *et al.* 2004). The native range of Glossocalyx is Southern Nigeria to Western Central Tropical Africa. The *Glossocalyx brevipes* has been documented in the treatment of diabetes and its interconnected diseases (Tsabang *et al.* 2016). Two important limonoids (1-deacetylkhivorin and 7-deacetylkhivorin) derived from Khaya grandifoliola (Meliaceae) (Bickii *et al.* 2000) also showed low binding energy. The Khaya grandifoliola, which is also known as African mahogany belongs to the family Meliaceae and is found abundantly in the Republic of Benin, Nigeria, the Democratic Republic of the Congo, Ivory Coast, Ghana, Guinea, Sudan, Togo, and Uganda. This plant has been utilized in polyherbal formulations for the treatment of T2DM (Kale *et al.* 2018).

Also, structure-based virtual screening of selected terpenoids with PTP1B revealed considerable interactions. The fact that the docking scores of the terpenoids were not comparable to that of the co-crystallized compound isothiazolidinone (a known PTP1B inhibitor) suggests that the terpenoids have lower affinity for PTP1B. The reference compound, with a lower docking score, demonstrates stronger binding, which is preferable in drug discovery. Although all the docked compounds had docking scores and MM-GBSA binding energy higher than those of the reference isothiazolidinone, the topmost compounds had favorable interactions with the binding pocket of the protein. The Beilshmiedic acid derivatives viz: Tsangibeilin B and Cryptobeilic acid C, which were isolated from Beilschmiedia cryptocaryoides (Lauraceae) (Talontsi *et al.* 2013) had the lowest binding score. Several bisnorterpenoids (isoiguesterin, 6-Oxoisoiguesterin, isoiguesterinol, and 20-Epi-isoiguesterinol) derived from *Ekebergia capensis* (Murata *et al.* 2008) also had low binding scores. In addition, galanolactone, a labdane diterpenoid derived from *Aframomum arundinaceum* (Zingiberaceae) (Wabo 2006) exhibited low binding energy. Van der Waals, nonpolar solvation, and Coulombs energy terms made the most significant contributions to the binding affinity of the ligands. The docking results guided the decision to focus on DPP4 as a therapeutic target, as the terpenoids showed more promising interactions with DPP4 compared to PTP1B. These findings suggest the potential utility of specific terpenoids in modulating DPP4 activity, which could be advantageous for diabetes management.

Based on our results from molecular docking and postdocking MM-GBSA which revealed DPP-4 as a more promising target, the top docked terpenoids were further subjected to ensemble docking analysis. Ensemble docking which involves the use of multiple molecular docking simulations was employed to improve the accuracy and reliability of predicting the top terpenoids binding mode and affinity with DPP-4 enzyme. To achieve this, a DPP-4 structure ensemble was constructed by subjecting the experimental structure to a 100-ns MD simulation which generated three clusters. Docking of the cluster representative structures against the hit triterpenoids (T1, T4, T2) along with the reference compound (alogliptin) showed that docking of top triterpenoids with the active site of DPP-4 conformational representatives was consistent with the earlier docking results. It is also worthy of note that interaction analysis of the docked conformation with representative clusters revealed that the amino acid interaction was preserved and reproducible.

The top terpene–DPP-4 complexes were subjected to MD simulation alongside the reference alogliptin and unliganded enzyme to capture a more comprehensive picture of biomolecular systems considering the inherent flexibility and dynamics of proteins. The MD simulation is often integrated with docking simulation and other molecular modeling techniques to further validate virtual screening through static docking and assess the stability of drug–receptor complexes. Computation of various thermodynamic parameters from the MD simulation trajectories revealed the structural stability and conformation flexibility of the ligand–DPP-4 complexes alongside the unliganded protein as depicted in Fig. 4. In the context of molecular modeling and structural biology, RMSD is often used to compare the structures of two molecules, such as a crystal structure and a MD simulation trajectory. To calculate RMSD, one first aligns the two sets of coordinates, typically by superimposing them using a least-squares fitting algorithm. Then, the distance between each corresponding pair of atoms in the two sets of coordinates is calculated, and the average of the squared distances is taken. Finally, the square root of the average squared distance is computed to obtain the RMSD value. The RMSD is a widely used metric in molecular modeling and structural biology, as it provides a quantitative measure of the similarity between two structures. A low RMSD value indicates a high degree of similarity, while a high RMSD value indicates a greater difference between the two structures. The RMSD values which indicate the overall structural stability of the DPP-4 enzyme back-bone C-α atoms in complex with alogliptin, T1, T4, and T2 were reported in this study. Except for the major fluctuations observed with the trajectory of DPP-4–cucurbitacin B complex, all the complexes exhibited relatively stable atomic trajectories displaying minor and consistent fluctuations. During MD simulation, low levels of RMSD values which are characterized by such consistent fluctuations indicate equilibration and stability of the system. Highly deviated structures after equilibration such as the observed RMSD of DPP-4–cucurbitacin B complex may imply major conformational transitions by DPP-4 to attain stable conformation of the enzyme with cucurbitacin B. The stability of drug–target complexes depends on their tendency of unfolding deviation from their initial structure. The unfolding tendency can be evaluated using various thermodynamic parameters including the RoG and SASA. In this study, the RoG values of the apoprotein and the protein–ligand complexes were maintained within 2 Å indicating stability of the molecular system. The RoG calculation helps to assess how spread out the mass of the complexes is from its rotation axis. It is often employed in MD simulation to estimate the compactness of the bound structures. The SASA is a measure of the total surface area of a molecule that is available for interaction with other molecules, such as enzymes or receptors. Our study indicate stability of the complexes as reported. It is calculated by measuring the area of the molecule that is exposed to solvent, such as water. The SASA is important because it can help predict the binding affinity of a molecule to its target. Generally, molecules with a larger SASA are more likely to bind strongly to their targets because they have more surface area available for interaction. Since the RoG values indicate compactness of the complex structures and those of SASA reflect the extent of the solvent-accessible surface tendency of the protein structure. Taken together, the terpene–DPP-4 complexes with reduced RoG and SASA values reflect a low tendency of unfolding deviation from their initial structure. Hydrogen bond number existing in the molecular systems was calculated in order to provide more insight into the stability of the protein–ligand complexes. The high number of H-bonds observed in the ligand–protein complexes further indicates well-bounded ligand and compact structures. Therefore, the stable intermolecular bonds characterized by the hydrogen bonds formed may account for the high tendency of the terpenoid–target complexes to maintain compact and well-folded biomolecular structure. The RMSF analysis often reveals how a portion of the protein structure deviates from its mean structure upon interactions with a ligand. Computation of the RMSF from the trajectory files helps to evaluate the crucial contributions that a protein’s amino acid residues make toward achieving stable conformations for protein–ligand complexes. In this study, the values of RMSF for DPP-4 enzyme residues toward the triterpenoids were calculated and shown in Fig. 4E. The plot demonstrates large variations in the amino acid residues between 220 and 260 as well as those around 680, with the RMSF reaching notable peaks. Other residues, in addition to these also feature some notable peaks. This suggests that they have a strong affinity for interacting with terpenoids and that the structures may be able to bind strongly to the protein at these points. The RMSF plot displays the degree of fluctuation seen for each protein residue, providing insight into the degree of adaptability they have in a changing environment. As a result, motifs or individual amino acid residues that exhibit higher RMSF and flexibility have a stronger propensity of interactions with the ligand.

The MM-GBSA calculation is often employed in molecular modeling investigations to assess the binding free energy of a compound with a receptor. It is often considered as a postprocessing technique for analyzing trajectories obtained from an MD simulation run. Given the limitations of molecular docking simulation, which is mostly reliant on MM calculations, the trajectories obtained from the MD simulations of the terpene–DPP-4 complexes were subjected to free energy simulation using MM-GBSA, which combines conventional MM with implicit solvation models. The main components of the free energy of the top complexes are van der Waals energy and electrostatic energy. Amino acids occurring around the binding pocket showed significant contributions to the overall binding affinity to the terpen–DPP-4 complexes as indicated by the MM-PBSA free energy decomposition analysis.

DFT is a quantum mechanical approach that calculates the electronic density of a system, which can then be used to calculate other properties such as total energy, electronic charge distribution, and molecular geometry. The essential chemical characteristics of the terpene structures were analyzed using frontier molecular orbital analysis of the completely optimized structures as shown in Table 6. According to FMO theory, one of the key factors that influence the biological activities of drug-like compounds is the energy level of their HOMO and LUMO orbitals (Hagar and Ahmed 2020). The HOMO energy indicates the compound’s ability to donate electrons, whereas the LUMO energy quantifies the compound’s capacity to accept electrons. All the triterpene structures displayed higher HOMO energy than the reference alogliptin, which is consistent with the molecular docking, ensemble docking, and postdocking MM-GBSA. In simple term, a compound with small energy gap is more polarizable and often associated with high reactivity (Olawale *et al.* 2021). These suggest that the hit compounds have high tendency of participating in molecular reactions. In a previous study, it was observed that clinically approved therapeutic showed good correlation with the experimental data having electron affinity value ranging between −1.5 and 2.0 eV (Matuszek and Reynisson 2016). The fact that all our hit compounds EA value lies within this range suggests a good druglikeness property. In addition, it was observed that the highest electrophilicity index was observed in cucurbitacin B, oxo-isoguesterin, and epi-isoguestrin. The electrophilicity index depicts information about electron system structure, stability, bonding, reactivity, and dynamics at ground and excited states (Ganesan *et al.* 2020). The authors further explained that compounds with a high electrophilicity index are more likely to interact with biomolecules, suggesting a preferential reactivity of these compounds. Overall, all the hit compounds show promising reactivity indices, which emphasizes significant potential as lead candidates.

The drug-likeness characteristics of the top triterpenoids were assessed to ascertain the suitability of the compounds as drug candidates. These compounds possess desirable properties (Table 7), which are important to the ultimate nutraceuticals and drug development. Using the Lipinski rule of five assessments, it was shown that all the hit compounds are in accordance to Lipinski rule. Based on Lipinski rule, orally active drugs have no more than one violation of the set criteria viz: less than five hydrogen bond donors, less than 10 hydrogen bond acceptors, molecular mass below 500 Da, and octanol-water partition coefficient <5 (Lipinski 2000, 2004). The fact that the compounds did not exceed one of the Lipinski violations suggests they can be readily absorbed and in therapeutic doses. The lead-likeness property was further corroborated by the Veber, Egan, and Muegge rule. Further emphasizing the druglikeness property of the drugs is the result from pain (Pan-assay interference compounds) alert prediction. While the previous rules focused on the oral bioavailability of the drug, pain alert helps to identify the tendency of the compounds to participate in nonspecific binding to multiple targets. The PAINS is one of the major results for false positive in drug development. Based on these predictions, it was observed that most of the top compounds do not contain scaffolds capable of nonspecific binding as such the high binding affinity obtained is less likely due to false positive. Furthermore, most of hits were not predicted as p-glycoprotein substrate. The Pgp is responsible for binding foreign particles and facilitating their rapid elimination from the body. Since most of the compounds are not pgp substrate, the implication is the compounds are likely to have long circulating time thereby achieving maximum therapeucity before clearance from the body.

The ultimate goal of computational modeling in natural product research is to rapidly predict, in advance of any targeted laboratory testing, novel biologically active compounds. Integrated molecular modeling methods applied in this study revealed several African-derived terpenoid structures with strong binding tendency to the DPP-4 and PTP1B enzymes. The docking results guided the decision to focus on DPP4 as a therapeutic target, as the terpenoids showed more promising interactions with DPP4 compared to PTP1B. Three hit triterpenoids viz: Cucurbitacin B, 20-Epi-isoiguesterinol, and 6-Oxoisoiguesterin were identified as promising inhibitors of DPP-4. Our *in silico* evidence revealed that these compounds maintained strong binding affinity and stable interactions tendency with DPP4 as indicated by ensemble docking, postdocking MM-GBSA, MD simulation, MD-based MM-GBSA, and DFT. The binding interactions of these triterpenoids were sustained by interactions with several amino acid residues in the catalytic triad, oxyanion cavity, hydrophobic S1 pocket, and charged S2 pocket in the active site region of DPP-4. In addition, the hit compounds exhibit good drug-likeness and acceptable ADMET properties. Although cucurbitacins have earlier been reported for antidiabetic activities, 6-Oxoisoiguesterin and 20-Epi-isoiguesterinol are less known for antidiabetic activity. Herbal formulations comprising infusion prepared from the leaves of Ekebergia capensis, a rich source plant of the reported bisnorterpenoids (20-Epi-isoiguesterinol, isoiguesterin, 6-oxoisoiguesterin, and isoiguesterinol) has been exploited and taken orally as antidiabetic agent for decades. The study has suggested bioactive agents from the selected herbs and further suggests molecular mechanism underpinning the antidiabetic properties of the hit triterpenoids, supporting the ethnopharmacological use of such African antidiabetic herbs. These findings may pave the way for the development of novel antidiabetic agents and nutraceuticals based on these promising *in silico* hits.

## Supplementary Material

vbae205_Supplementary_Data
