# Supplementary material for: Identification of promising dipeptidyl peptidase-4 and protein tyrosine phosphatase 1B inhibitors from selected terpenoids through molecular modeling
Source: Bioinform Adv. 2024 Dec 19;5(1):vbae205. doi: 10.1093/bioadv/vbae205 (PMC11751579; doi:10.1093/bioadv/vbae205)
Supplement: vbae205_Supplementary_Data [file vbae205_supplementary_data.zip › f933b_Supplementary materials.docx]

Identification of promising dipeptidyl peptidase-4 and protein tyrosine phosphatase 1B inhibitors from selected terpenoids through molecular modeling

**Table S1: Top docking terpenes against DPP4 and PTP1B**

| Compounds | Class | Source plants | DPP4  (Kcal/mol) | PTPB1  (Kcal/mol) |
| --- | --- | --- | --- | --- |
| Alogliptin (S1) |  |  | -7.7 |  |
| Isothiazolidinone (S2) |  |  |  | -9.8 |
| 3- Benzoylhosloppone | Abietane diterpenes | *Hoslundia opposita (*Lamiaceae*)* | -10.0 | -8.0 |
| 20-*Epi*-isoiguesterinol | Bisnorterpenes | Bisnorterpenes | -9.9 | -7.1 |
| Cucurbitacin B | Pentacyclic triterpenes | *Cogniauxia podolaena (*Cucurbitaceae*)* | -9.9 | -6.4 |
| Isoiguesterin | Bisnorterpenes | Bisnorterpenes | -9.7 | -7.6 |
| Cryptobeilic acid C | Beilshmiedic acid derivatives | *Beilschmiedia cryptocaryoides (*Lauraceae*)* | -9.5 | -8.6 |
| Ent-18-*E*-Caffeoyloxy-7 -hydroxy-3-cleroden-15-oic acid | Clerodane and labdane diterpenoids | *Nuxia sphaerocephala (*Loganiaceae*)* | -9.4 | -8.0 |
| 6-Oxoisoiguesterin | Bisnorterpenes | *Bisnorterpenes* | -9.3 | -7.5 |
| **Liriodenine** | Acyclic triterpenes | *Ekebergia capensis (*Zingiberaceae*)* | -9.2 | -7.7 |
| Isoiguesterinol | Bisnorterpenes | *Bisnorterpenes* | -9.0 | -7.1 |
| 1-Deacetylkhivorin | Limonoids | *Khaya grandifoliola (Meliaceae)* | -8.9 | -5.6 |
| Cucurbitacin D | Pentacyclic triterpenes | *Cogniauxia podolaena (*Cucurbitaceae*)* | -8.9 | -7.0 |
| Ent-18-*E*-Caffeoyloxy-8(17)-labden-15-oic acid | Clerodane and labdane diterpenoids | *Nuxia sphaerocephala (*Loganiaceae*)* | -8.8 | -7.0 |
| 7-Deacetylkhivorin | Limonoids | *Khaya grandifoliola (*Meliaceae*)* | -8.8 | -5.4 |
| Gedunin | Limonoids | *Khaya grandifoliola (*Meliaceae*)* | -8.7 | -6.0 |
| Tsangibeilin B | Beilshmiedic acid derivatives | *Beilschmiedia cryptocaryoides (*Lauraceae*)* | -8.6 | -8.5 |
| Oleanolic acid | Pentacyclic triterpenes | *Nuxia sphaerocephala (*Loganiaceae*)* | -8.6 | -6.1 |
| (13*S*)-Ent-18-*E*-Coumaroyloxy-8(17)-labden-15-oic acid | Clerodane and labdane diterpenoids | *Nuxia sphaerocephala (*Loganiaceae*)* | -8.5 | -7.4 |
| Ekeberin C1 | Limonoids | *Ekebergia capensis (*Zingiberaceae*)* | -8.5 | -6.2 |
| 24-Methylene cycloartenol | Pentacyclic triterpenes | *Entandrophragma angolense (*Meliaceae*)* | -8.5 | -7.1 |
| 3-Friedelanone | Pentacyclic triterpenes | *Hypericum lanceolatum (*Hypericaceae*)* | -8.5 | -6.6 |
| Lupeol | Pentacyclic triterpenes | *Hymenocardia acida (*Phyllanthaceae*)* | -8.3 | -5.2 |
| 3-Oxolupenal (3-oxolup-20(29)-en-30-al) | Pentacyclic triterpenes | *Nuxia sphaerocephala (*Loganiaceae*)* | -8.2 | -7.0 |
| 3-Oxolupenol (30-hydroxylup-20(29)-en-3-one) | Pentacyclic triterpenes | *Nuxia sphaerocephala (*Loganiaceae*)* | -8.2 | -6.2 |
| Hydroxyvernolide | Sesquiterpenes and sesquiterpene lactones | *Vernonia amygdalina (*Asteraceae*)* | -8.1 | -5.5 |
| Pristimerin | Pentacyclic triterpenes | *Maytenus senegalensis (*Celastraceae*)* | -8.1 | -7.5 |
| 3 -Hydroxylupenal (3 -hydroxylup-20(29)-en-30-al) | Pentacyclic triterpenes | *Nuxia sphaerocephala (*Loganiaceae*)* | -8.1 | -7.0 |
| Vernolide | Sesquiterpenes and sesquiterpene lactones | *Vernonia amygdalina (*Asteraceae*)* | -8.0 | -5.5 |
| 3-*O*-betulinic acid *p*-coumarate | Pentacyclic triterpenes | *Baillonella toxisperma (*Sapotaceae*)* | -8.0 | -5.2 |
| 2 ,3 ,19 -Trihydroxy-urs-12-20-en-28-oic acid | Pentacyclic triterpenes | *Kigelia africana (Bignoniaceae)* | -8.0 | -5.4 |
| Ferruginol | Abietane diterpenes | *Fuerstia africana (Lamiaceae)* | -7.9 | -7.6 |
| Betulinic acid | Pentacyclic triterpenes | *Entandrophragma angolense (*Meliaceae*)* | -7.9 | -5.6 |
| Galanolactone | Clerodane and labdane diterpenoids | *Aframomum arundinaceum (*Zingiberaceae*)* | -7.8 | -6.5 |
| Artemisinin | Sesquiterpenes and sesquiterpene lactones | *Artemisia annua (*Asteraceae*)* | -7.8 | -5.9 |
| Cryptobeilic acid A | Beilshmiedic acid derivatives | *Beilschmiedia cryptocaryoides (*Lauraceae*)* | -7.8 | -6.7 |
| Caesaldekarin C | Cassane furanoditerpenes | *Caesalpinia volkensii (*Leguminosae*)* | -7.7 | -5.5 |
| Tagitinin C | Sesquiterpenes and sesquiterpene lactones | *Tithonia diversifolia (*Asteraceae*)* | -7.7 | -5.9 |
| Cryptobeilic acid D | Beilshmiedic acid derivatives | *Beilschmiedia cryptocaryoides (*Lauraceae*)* | -7.7 | -7.0 |
| Ent-7 Hydroxy-2-oxo-3-cleroden-15-oic acid | Clerodane and labdane diterpenoids | *Nuxia sphaerocephala (*Loganiaceae*)* | -7.6 |  |
| Ent-2,7-Dioxo-3-cleroden-15-oic acid | Clerodane and labdane diterpenoids | *Nuxia sphaerocephala (*Loganiaceae*)* | -7.6 |  |
| Vernodalin | Sesquiterpenes and sesquiterpene lactones | *Vernonia amygdalina (*Asteraceae*)* | -7.6 |  |
| Cryptobeilic acid B | Beilshmiedic acid derivatives | *Beilschmiedia cryptocaryoides (*Lauraceae*)* | -7.6 |  |
|  | Acyclic triterpenes | *Ekebergia capensis (*Zingiberaceae*)* | -7.5 |  |
| Dehydrobrachylaenolide | Sesquiterpenes and sesquiterpene lactones | *Dicoma anomala subsp. gerrardii (*Asteracea*e)* | -7.4 |  |
| (13*S*)-Ent-7 -Hydroxy-3-cleroden-15-oic acid | Clerodane and labdane diterpenoids | *Nuxia sphaerocephala (*Loganiaceae*)* | -7.3 |  |
| Methylangolensate | Limonoids | *Khaya grandifoliola (*Meliaceae*)* | -7.3 |  |
| 7 -Acetoxy-6,12-dihydroxy-abieta- 8,12-Diene-11,14-dione | Abietane diterpenes | *Plectranthus hadiensis (*Lamiaceae*)* | -7.3 |  |
| 16,17- Dihydrobrachycalyxolide | Sesquiterpenes and sesquiterpene lactones | *Vernonia brachycalyx (*Asteraceae*)* | -7.3 |  |
| Aulacocarpin A | Clerodane and labdane diterpenoids | *Aframomum zambesiacum (*Zingiberaceae | -7.2 |  |
| 13 -*Epi*-dioxiabiet-8(14)-en-18-ol | Abietane diterpenes | *Hyptis suaveolens (*Lamiaceae*)* | -7.1 |  |
| Vernodalol | Sesquiterpenes and sesquiterpene lactones | *Vernonia amygdalina (*Asteraceae*)* | -7.1 |  |
| Ajugarin-1 | Sesquiterpenes and sesquiterpene lactones | *Ajuga remota (*Lamiaceae*)* | -7.1 |  |
| 11 -Hydroxymuzigadiolide | Coloratane sesquiterpenes | *Warburgia ugandensis (*Canellaceae*)* | -7.1 |  |
| Aulacocarpin A | Clerodane and labdane diterpenoids | *Aframomum zambesiacum (*Zingiberaceae | -7.0 |  |
| 3-Deoxyaulacocarpin A | Clerodane and labdane diterpenoids | *Aframomum zambesiacum (*Zingiberaceae | -7.0 |  |
| Galanal B | Clerodane and labdane diterpenoids | *Aframomum arundinaceum (*Zingiberaceae*)* | -7.0 |  |
| Urospermal A-15-*O*-acetate | Sesquiterpenes and sesquiterpene lactones | *Dicoma tomentosa (*Asteraceae | -7.0 |  |
| Okundoperoxide | Sesquiterpenes and sesquiterpene lactones | *Scleria striatinux (*Cyperaceae*)* | -7.0 |  |
| (*E)*-8(17), 12-labddiene-15,16-dial | Clerodane and labdane diterpenoids | *Aframomum latifolium* | -6.9 |  |
| Galanal A | Clerodane and labdane diterpenoids | *Aframomum* | -6.8 |  |
| Cinnamolide-3 -acetate | Coloratane sesquiterpenes | *Warburgia ugandensis (*Canellaceae*)* | -6.8 |  |
| Oplodiol | Sesquiterpenes and sesquiterpene lactones | *Reneilmia cincinnata (*Zingiberaceae*)* | -6.7 |  |
| Vernangulide A | Sesquiterpenes and sesquiterpene lactones | *Vernonia angulifolia (*Asteraceae*)* | -6.7 |  |
| (*E*) Labda-8,12-diene-15,16 dial | Clerodane and labdane diterpenoids | *Aframomum arundinaceum (*Zingiberaceae*)* | -6.6 |  |
| Muzigadial | Coloratane sesquiterpenes | *Warburgia ugandensis (*Canellaceae) | -6.4 |  |
| Cinnamolide | Coloratane sesquiterpenes | *Warburgia ugandensis (*Canellaceae*)* | -6.4 |  |
| Ugandensidial | Coloratane sesquiterpenes | *Warburgia ugandensis (Canellaceae)* | -6.3 |  |
| Mukaadial | Coloratane sesquiterpenes | *Warburgia ugandensis (*Canellaceae*)* | -6.2 |  |
| 4(13),7-Coloratadiene-12,11-olide | Coloratane sesquiterpenes | *Warburgia ugandensis (*Canellaceae*)* | -6.1 |  |
| methyl-14,15-epoxylabda-8(17), 12(*E*)-Diene-16-oate | Clerodane and labdane diterpenoids | *Turreanthus africanus (*Meliacea*e)* | -5.3 |  |
| Coranarin B | Clerodane and labdane diterpenoids | *Aframomum* | -3.5 |  |
| Coronarin B | Clerodane and labdane diterpenoids | *Aframomum latifolium* | -2.0 |  |
| 11-Hydroxy-19-(4-hydroxy-benzoyloxy)-abieta -5,7,9(11),13-tetraene-12-one | Abietane diterpenes | *Plectranthus purpuratus (*Lamiaceae*)* | -2.0 |  |
| 11-Hydroxy-19-(3,4-dihydroxybenzoyloxy)- abieta-5,7,9(11),13-tetraene-12-one | Abietane diterpenes | *Plectranthus purpuratus (*Lamiaceae*)* | -2.0 |  |
| 6 ,9 -Dihydroxy-4(13),7- coloratadiene- 11,12-dial | Coloratane sesquiterpenes | *Warburgia ugandensis (*Canellaceae*)* | -2.0 |  |
| 22-Hydroxyhopan-3-one | Pentacyclic triterpenes | *Cassia siamea (*Fabaceae*)* | -2.0 |  |
| 11-Hydroxy- 19-(methyl-buten-2-oyloxy)-abieta -5,7,9 (11),13-tetraene-12-one | Abietane diterpenes | *Plectranthus purpuratus (*Lamiaceae*)* | -1.5 |  |
| Ent-15-*E*-Caffeoyloxy-8(17)-labden-18-oic acid | Clerodane and labdane diterpenoids | *Nuxia sphaerocephala (*Loganiaceae*)* | -1.2 |  |
| Aframodial | Clerodane and labdane diterpenoids | *Aframomum latifolium* | -1.2 |  |
| (*E*)-15,15-diethoxylabda-8(17),12-dien-16-al | Clerodane and labdane diterpenoids | *Aframomum latifolium* | -1.2 |  |
| 16-Oxolabda-8(17),12(*E*)-dien- 15-oic acid | Clerodane and labdane diterpenoids | *Turreanthus africanus (*Meliaceae*)* | -1.2 |  |
| Zambesiacolactone A | Clerodane and labdane diterpenoids | *Turreanthus africanus (*Meliaceae*)* | -1.2 |  |
| Zambesiacolactone B | Clerodane and labdane diterpenoids | *Aframomum zambesiacum (*Zingiberaceae *)* | -1.2 |  |
| Methyl uguenesonate | Limonoids | *Vepris uguenensis (*Rutaceae*)* | -1.2 |  |
| 6-Acetylswietenolide | Limonoids | *Khaya grandifoliola (*Meliaceae*)* | -1.2 |  |
| - Acetoxydihydronomilin | Limonoids | *Entandrophragma angolense (*Meliaceae | -1.2 |  |
| 7-Deacetoxy-7-oxogedunin | Limonoids | *Ekebergia capensis (*Zingiberaceae*)* | -1.2 |  |
| Ekeberin C3 | Limonoids | *Ekebergia capensis (*Zingiberaceae*)* | -1.2 |  |
|  | Acyclic triterpenes | *Ekebergia capensis (*Zingiberaceae*)* | -1.2 |  |
| 5*E*,10(14)-germacradien-1 ,4 -diol | Sesquiterpenes and sesquiterpene lactones | *Reneilmia cincinnata (*Zingiberaceae*)* | -1.2 |  |
| 15-Acetoxy-8-[(2-methylbutyryloxy)]- 14-oxo-4,5-cis-acanthospermolide | Sesquiterpenes and sesquiterpene lactones | *Acanthospermum hispidum (*Asteraceae*)* | -1.2 |  |
| Vernangulide B | Sesquiterpenes and sesquiterpene lactones | *Vernonia angulifolia (*Asteraceae*)* | -1.2 |  |
| 3-Hydroxy-20(29)-lupen-28-ol | Pentacyclic triterpenes | *Schefflera umbellifera (*Araliaceae*)* | -1.2 |  |
| 20-*Epi*bryonolic acid | Pentacyclic triterpenes | *Cogniauxia podolaena (*Cucurbitaceae*)* | -1.2 |  |

Table S2: Post-docking MMGBSA scores of top terpenes against DPP-4 and PTP1B

| S/N | Compounds | MMGBSA Bind | Bind Coulomb | Covalent | Hbond | Bind Lipo | Bind Packing | Bind Solv GB | Bind vdW |
| --- | --- | --- | --- | --- | --- | --- | --- | --- | --- |
| DPP-4 | | | | | | | | | |
| S1 | Alogliptin | -37.02 | -8.32 | 1.758 | -2.305 | -14.135 | -2.882 | 28.224 | -39.358 |
| T1 | Cucurbitacin B (169) | **-47.80** | -35.29 | 11.264 | -3.363 | -19.397 | 0 | 57.773 | -58.782 |
| T2 | 20-*Epi*-isoiguesterinol (101) | -29.78 | -19.15 | 0.991 | -2.029 | -23.209 | -0.795 | 54.704 | -40.289 |
| T3 | Isoiguesterin (100) | -28.75 | -10.864 | 1.379 | -0.883 | -23.836 | -0.785 | 46.43 | -40.191 |
| T4 | 6-Oxoisoiguesterin (103) | -32.90 | -17.498 | 0.971 | -1.881 | -22.559 | -0.867 | 46.477 | -37.532 |
| T5 | Liriodenine (106) | **-39.11** | -7.721 | 1.263 | -0.708 | -17.902 | -5.46 | 23.842 | -32.426 |
| T6 | Isoiguesterinol (102) | -29.36 | -16.714 | 2.695 | -1.903 | -21.807 | -0.776 | 50.067 | -40.923 |
| T7 | 1-Deacetylkhivorin (92) | -19.72 | -15.43 | 5.832 | -2.132 | -15.078 | -1.142 | 44.856 | -36.626 |
| T8 | 7-Deacetylkhivorin (91): | -25.71 | 2.965 | 2.832 | -1.306 | -14.321 | -2.303 | 34.239 | -47.818 |
| PTP1B | | | | | | | | | |
| S2 | ISOTHIAZOLIDINONE | -85.23 | -99.344 | 4.973 | -6.852 | -20.154 | -3.171 | 92.36 | -53.038 |
| T9 | Tsangibeilin B (153) | -42.32 | -26.194 | 1.513 | -2.345 | -28.259 | -1.313 | 51.722 | -37.444 |
| T10 | Cryptobeilic acid C (151) | -44.09 | -33.452 | 0.954 | -2.304 | -27.212 | -1.285 | 57.253 | -38.042 |
| T5 | Liriodenine (106) | -34.39 | -1.887 | 0.367 | -0.871 | -20.387 | -6.538 | 28.248 | -33.322 |
| T3 | Isoiguesterin (100) | -42.07 | -21.819 | 5.568 | -2.717 | -22.009 | -2.469 | 31.443 | -30.066 |
| T4 | 6-Oxoisoiguesterin (103) | -41.11 | -17.819 | 5.163 | -2.82 | -20.149 | -2.49 | 28.487 | -31.482 |
| T6 | Isoiguesterinol (102 | -45.82 | -31.997 | 6.987 | -3.171 | -21.847 | -2.484 | 35.587 | -28.891 |
| T11 | Galanolactone (85) | -43.56 | -38.903 | 4.492 | -2.116 | -19.293 | 0 | 40.361 | -28.1 |
| T2 | 20-*Epi*-isoiguesterinol (101) | -29.06 | -22.418 | 4.903 | -2.383 | -16.142 | -2.405 | 36.822 | -27.439 |
